# Modelling the rate of trainees transitioning to Fellowship before achieving competence under the RANZCP’s Alternative Assessment Pathway to the Objective Structured Clinical Examination

**DOI:** 10.1177/10398562231202120

**Published:** 2023-09-15

**Authors:** Andrew Amos, Michael James Weightman, Edward Miller

**Affiliations:** College of Medicine and Dentistry, 104397 James Cook University, Townsville, Australia; School of Medicine, 1066 The University of Adelaide, Adelaide, SA, Australia;; College of Medicine and Public Health, 1065 Flinders University, Bedford Park, SA, Australia; Division of Psychological Medicine, Faculty of Medical & Health Science, 1415 University of Auckland, Auckland, New Zealand

**Keywords:** psychiatric training, medical education, professional ethics

## Abstract

**Objective:**

The Objective Structured Clinical Examination (OSCE) has been removed from the Royal Australian and New Zealand College of Psychiatrists’ (RANZCP) training pathway. This decision occurred in the context of an Alternative Assessment Pathway (AAP) necessitated by Covid-19, justified by logistical, methodological and equity concerns. The false positive rate of trainees progressing to Fellowship before achieving competence is a key indicator for evaluating any assessment leading to psychiatric Fellowship. Variations in the statistical properties of the AAP and OSCE were analysed for their impact on pre-competent trainees progressing to Fellowship.

**Method:**

Starting with the false positive scenario presented to justify discontinuing the OSCE, false positive rates associated with the AAP and OSCE were calculated based on different assumptions about reliability and accuracy.

**Results:**

The analyses suggest that less reliable and less accurate alternatives to the OSCE, such as the AAP, increase the number of pre-competent trainees progressing to Fellowship.

**Conclusions:**

Given possible increases in pre-competent trainees progressing to Fellowship while alternatives to the OSCE are finalised, confidence in the RANZCP's training program demands robust public analyses of those alternatives.

The Royal Australian and New Zealand College of Psychiatrists’ (RANZCP) training program was redesigned to a competency-based format in 2012.^1^ Existing mandatory assessments were combined with workplace-based assessments to drive and evaluate the acquisition of sufficient knowledge, skills and attributes for certification of competent independent psychiatric practice. Before the Covid-19 pandemic, broad concern about the program of assessments, particularly persistently low marks on the Essay-Style Examination, stimulated a review by the Australian Council for Educational Research (ACER).^2^ Practical and theoretical concerns raised by the pandemic led to a further review in 2022^3^ and the decision to drop the Objective Structured Clinical Examination (OSCE) from the RANZCP training pathway in 2023.^4,5^ One broader consideration was the Australian Medical Council's (AMC) advice to all medical colleges to move away from high-stakes exams.^6^ This article considers arguments concerning this decision and models the false positive rate of the Alternative Assessment Pathway (AAP) given different assumptions about reliability and accuracy.

## Rethinking the Objective Structured Clinical Examination in the face of the pandemic

The OSCE has been the key high-stakes assessment of clinical skills in the RANZCP training pathway since replacing the Observed Clinical Interview (OCI) long case in 2012. The structured format, standardised marking and multiple items increase reliability and content coverage over the long case.^7^ While ACER described the traditional OSCE as fit-for-purpose in 2020,^2^ the impossibility of staging the OSCE as usual during the Covid-19 pandemic and the subsequent failed delivery of a large-scale online version led to its abandonment. As an interim replacement, the AAP was developed to allow trainees for whom the OSCE was the only remaining mandatory assessment to progress to Fellowship. This was approved by the RANZCP Board^4^ and the AMC.^8^ The AAP included a Portfolio Review (PR) that considered performance across the three most recent In-Training Assessments (ITAs), previously designed to assess whether a trainee had successfully completed a training rotation. If the candidate did not pass the PR, they were required to sit a centrally administered Case-based Discussion (CbD).^4^

A modified Clinical Competency Assessment (CCA) format and an Integrated Assessment Pathway have been considered as OSCE replacements.^9^ Both involve longitudinal assessment with broad sampling of clinical skills, with multiple supervisors using formal and informal observation. They promote feedback and documentation which can be directly linked with a coherent record of learning outcomes. This follows trends in medical education against high-stakes exams and recognition that assessments embedded in workplace tasks have increased ecological validity over standardised patient exams.^10^ Compared with the OSCE, they may better measure competencies sometimes misnamed ‘soft skills’, such as communication, collaboration, advocacy and cultural competence.

Despite these potential advantages, a recent petition to the RANZCP highlighted widespread concern about removing the OSCE from the RANZCP training pathway.^9^ The OSCE has broad acceptance within the medical community and has the ancillary benefit of encouraging candidates to spend a dedicated amount of time in structured, collaborative study throughout their preparation. Furthermore, the stressful, challenging public health environment in which training is conducted may not be conducive to learning or summative assessment. Supervisor training and standardisation is an acknowledged problem, alongside fears that role conflicts may adversely affect the supervisor relationship. Most importantly, the significantly higher pass rate for candidates under the AAP have been interpreted as prima facie evidence of a lower standard of assessment.

## Detecting pre-competent trainees with the Objective Structured Clinical Examination and Alternative Assessment Pathway

The announcement of the OSCE’s removal from the RANZCP's training pathway suggested the priority should be minimising the false positive rate of pre-competent candidates assessed as competent.^4,5^ It suggested the AAP's performance would match the OSCE but did not consider the potential for false positive results during the first phase (the Portfolio Review). In addition, it calculated the AAP false positive rate using raw figures, while it estimated the OSCE false positive rate by making assumptions about probability distributions. Most problematic, the original false positive analysis based the OSCE estimate on the 95% confidence interval, which represents confidence whether the population mean lies within the interval based on the sample, not the proportion of non-competent candidates who pass the exam due to chance variation. This aim of this article is to re-examine the possibility that the AAP would be less effective at detecting pre-competent trainees than the OSCE using more realistic assumptions.

## Methods

Assessing competency-based training relies on differentiating two populations: a cohort that has achieved competence and a cohort that has not achieved competence (i.e. non- or pre-competent).^11^ For example, junior doctors entering psychiatric specialty training are systematically different from psychiatric trainees entering Fellowship, and the utility of any putative assessment of psychiatric competence is determined by how well it can differentiate between the two.

This article re-analysed the false positive rate of the AAP reported by Schuwirth and the RANZCP Communique^3,4^ using the assumption of a normal distribution of competence and showing the effect of unstated assumptions about the PR.

## Results

Consistent with Schuwirth’s analysis,^3,4^ OSCE/AAP result distributions were assumed normal. Further, the mean competent and non-competent cohort scores were assumed to be 100 and 50, respectively, both with standard deviation (SD) 15. Combining figures reported by Schuwirth and the end-of-year training report for 2022^12^ provided the number of candidates, the number who passed/failed the PR and the number who passed/failed the CbD (Table 1). OSCE pass rates were taken from 2019, the last year unaffected by the pandemic.^13^

**Table 1.** Pass/fail rates for the objective structured clinical examination and interim assessment pathway between 2016 and 2022

Assessment	Total:	Pass (%)	Fail (%)	Total:	Pass (%)	Fail (%)
Assessment pathway 2022	444 (Portfolio Review)	397 (89%)	47 (11%)	47 (Case-based Discussion)	23 (48%)	24 (52%)
Objective structured clinical examination 2019	299	206 (69%)	93 (31%)			
**Pass rate for Objective structured clinical examination/Assessment pathway 2016–2022**						
2016	2017	2018	2019	2020	2021	2022
71%	66%	72%	69%	N/-A	N/A	90%

**Figure 1.**
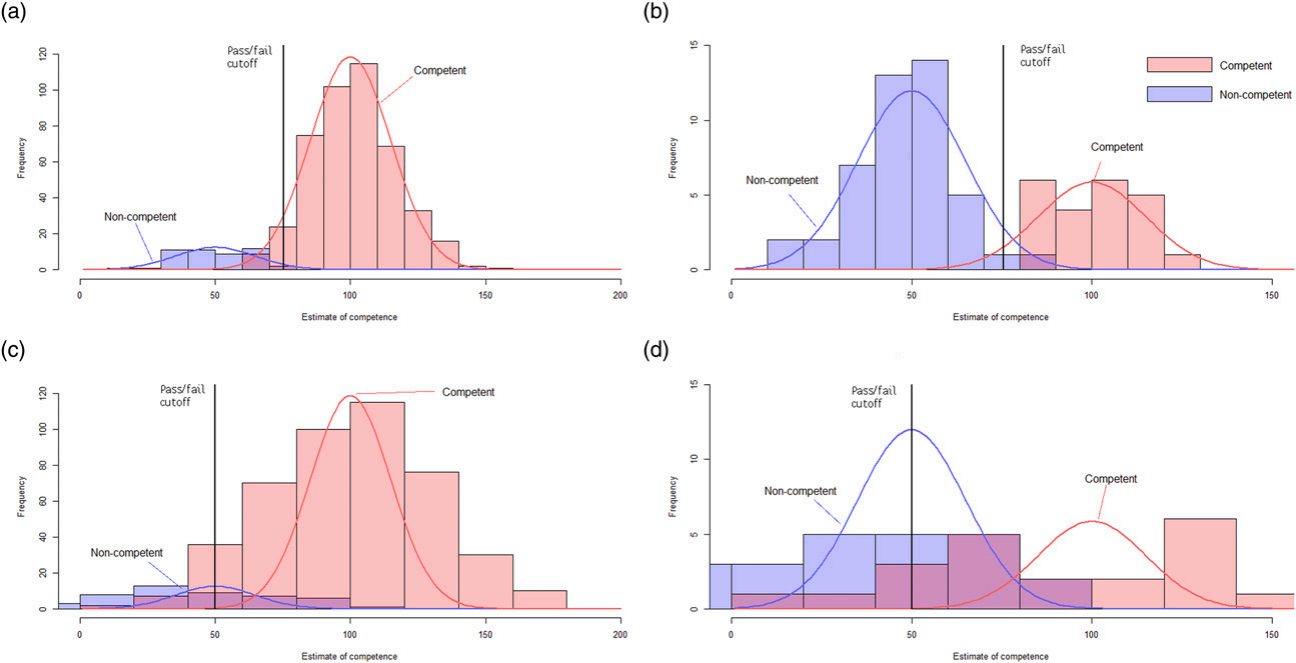
False positive rates across the two stages of the Assessment pathway. (a) Stage one - Portfolio Review - Low SD. (b) Stage Two - Case-based Discussion - Low False Positive. (c) Stage one - Portfolio Review - High SD. (d) Stage Two - Case-based Discussion - High False Positive

**Figure 2.**
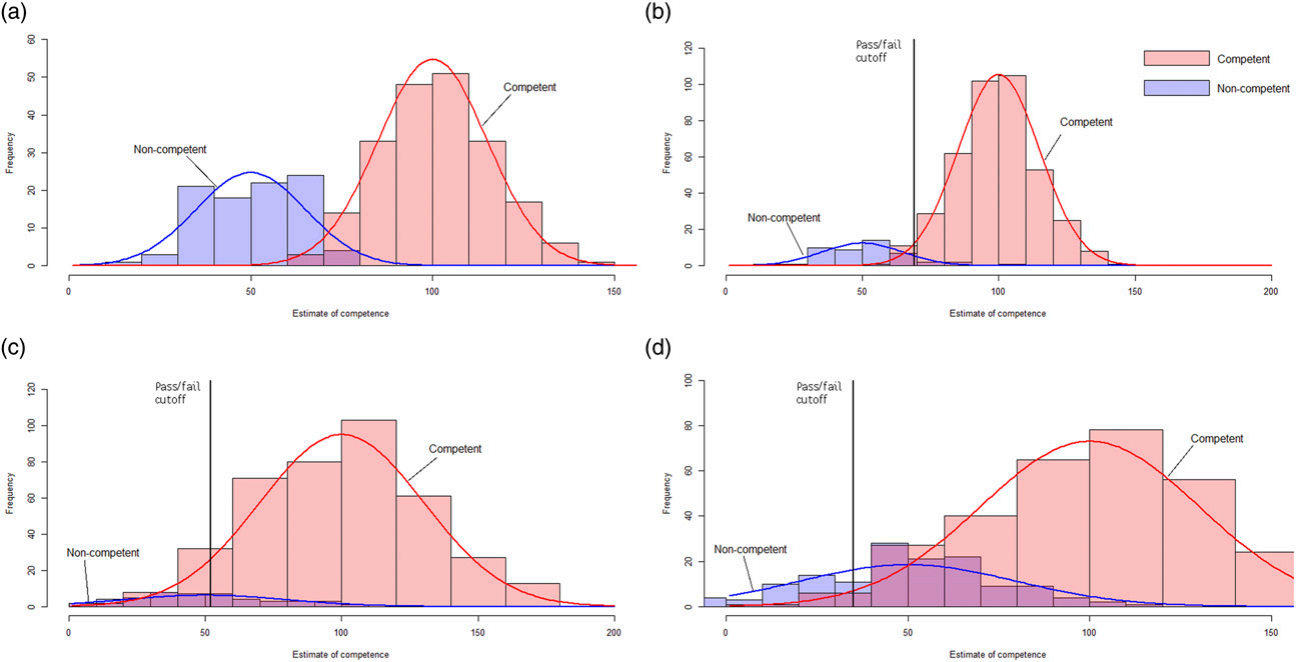
Combined AAP false positive rates comparing different assumptions about reliability and accuracy. (a) Objective structured clinical examination 2019. (b) Assessment pathway 2022 - High reliability, low fail rate. (c) Assessment pathway 2022 - Low reliability, low fail rate. (d) Assessment pathway 2022 - Low reliability, high fail rate.

Probability distributions were generated from these figures to show the impact of assumptions about the PR and CbD. Figures 1 and 2 show probability distributions as red and blue lines (representing competent and non-competent cohorts, respectively) superimposed on randomly generated representative histograms (also in red and blue).

Figure 1(a), (b) show that assuming highly reliable PR/CbD minimises false positives across both stages. Figure 1(c), (d) show that if the PR/CbD are less reliable than the OSCE, the competence scores measured by both stages overlap to a large degree, forcing lower cut-offs, and increasing false positives in both stages.

The pass rate of the March 2022 AAP was much higher than the pass rate of the 2016–2019 OSCEs (Table 1). Additional distributions were modelled to illustrate the impact on false positives of assuming that passing the AAP did not assert the same level of competence as the 2019 OSCE. Figure 2(a) shows the distribution for the 2019. Figure 2(b)–(d) show the effect of varying the assumptions that (b) the 2022 AAP was reliable, with a low fail rate; (c) the 2022 AAP was less reliable, with a low fail rate; (d) the 2022 AAP was less reliable, with a high fail rate.

Table 2 shows the impact of variations in the assumptions about the properties of the PR distribution on the likely number of false positives generated by the AAP.

**Table 2.** False positive rates for Portfolio Review under different assumptions

Royal Australian and New Zealand College of Psychiatrists Communique	Reliable/Low fail scenario	Unreliable/Low fail scenario	Unreliable/High fail scenario
0/397 (0%)	6/397 (1.5%)	22/397 (5.5%)	106/305 (34.7%)

## Discussion

Prior to the pandemic, the OSCE was a highly familiar part of the training pathway accepted as a valid measure of competence and tool of assessment-driven learning. Removing the OSCE from the training pathway before establishing and validating an alternative means of assessing the core clinical skills required for competent practice as an independent psychiatrist has generated significant discussion amongst RANZCP members. Our analyses show that if replacement(s) for the OSCE are less accurate and reliable, they may increase the number of pre-competent trainees transitioning to Fellowship.^4^

The OSCE provided a standardised, reliable and accurate estimate of a broad range of clinical skills. Taken at face value, the much higher pass rate associated with the AAP suggests that it asserts a lower standard of competence. In addition, the AAP’s lack of standardised content and use of untrained assessors suggests it was a much less reliable measure of individual clinical competence than the OSCE.

Our analyses demonstrate that the adequacy of OSCE substitutes depend upon the statistical properties of the constituent assessments. If the AAP less reliably asserted a lower standard of competence than past OSCEs, the rate of trainees progressing to Fellowship before achieving competence is likely to have increased. It appears broadly accepted that the pandemic forced trade-offs between the feasibility, fairness and statistical robustness of RANZCP assessments, and that the assessment decisions based on the AAP should be accepted as the most valid and reliable available in the circumstances. Nevertheless, we suggest that robust analyses of the AAP and future alternatives to the OSCE are made publicly available. These analyses must describe the nature and extent of the trade-offs necessary to retain public confidence in the integrity of the RANZCP's training pathway.

In our opinion, while it is possible that there are alternatives to the OSCE that have greater reliability and validity, it is risky to abandon the current gold standard before those other methods are finalised. Even if it is assumed that some combination of low-stakes assessments could at some future time approach the reliability and validity of the OSCE, there is no reason that an OSCE could not be included as a component of a programmatic assessment of clinical competence until it was demonstrated that it could be effectively replaced. The forthcoming review of the CEQ and MEQ is an opportunity to reconsider whether the evidence provided by an OSCE is sufficiently different from all other summative assessments to justify its reintroduction to the curriculum.^14^ We note reintroduction seems unlikely at this point given the opposition of the RANZCP and AMC.

## Limitations

This article explores the implications of different assumptions about the reliability and accuracy of the AAP for the false positive rate of candidates with a lower than acceptable level of competence achieving a passing grade. As the OSCE has been abandoned without specifying concretely what system of assessment will replace it, this limitation appears to be unavoidable. Finally, the limited data available meant it was impossible to analyse potential confounds such as the unusually large cohort of candidates taking the AAP for the first time compared with OSCE cohorts, the relative delay in undergoing assessment and the reliance upon formative instead of summative assessments to judge competence. It is likely that there were multiple confounds; for example, the larger number of first-time candidates may have increased the probability of passing as it is known that candidates resitting OSCEs pass at lower rates, while entering the AAP later in training may have increased the probability as candidates have accumulated more clinical experience.

## Conclusions

In the absence of a confirmed final alternative model of assessment, the loss of the OSCE from the RANZCP’s training pathway means that it is not currently possible to confidently estimate how likely it is that current trainees in Australia and New Zealand will have demonstrated a comparable level of competence before they achieve Fellowship. Our analyses demonstrate how the actual AAP used during the pandemic may have significantly elevated false positive rates compared to past OSCEs. Even if it is accepted that there are drawbacks to the OSCE, the decision to abandon it in the absence of a readily available and robust alternative appears risky.
